# Functional Amyloidogenesis and Cytotoxicity—Insights into Biology and Pathology

**DOI:** 10.1371/journal.pbio.1001459

**Published:** 2012-12-27

**Authors:** Douglas M. Fowler, Jeffery W. Kelly

**Affiliations:** 1 Department of Genome Sciences, University of Washington, Seattle, Washington, United States of America; 2 Department of Chemistry, The Scripps Research Institute, La Jolla, California, United States of America; 3 Department of Molecular and Experimental Medicine, The Scripps Research Institute, La Jolla, California, United States of America; 4 Skaggs Institute for Chemical Biology, The Scripps Research Institute, La Jolla, California, United States of America

## Abstract

Prions are self-templating protein structures that can be transferred from organism to organism. The [Het-s] prion propagates as a functional amyloid aggregate in the filamentous fungi *Podospora anserina*, and is involved in mediating heterokaryon incompatibility. Fusion of a *P. anserina* strain harboring the [Het-s] prion with another strain expressing the soluble Het-S protein results in cell death. The mechanism of Het-s/Het-S-mediated cell death has now been revealed in a paper just published in *PLOS Biology*. The study shows that Het-s and Het-S C-terminal domain co-amyloidogenesis induces a profound conformational rearrangement in the N-terminal Het-S HeLo domain, resulting in the exposure of a nascent transmembrane helix. Oligomerization of these helices leads to pore formation, leakage of the cytosolic contents, and subsequent cell death. Thus, Het-s amyloid plays a major role in the life cycle of *P. anserina* by orchestrating a complex conformational change in the Het-S protein, resulting in cytotoxicity by compromising membrane integrity. This ability of Het-s functional amyloid to initiate programmed cytotoxicity by mediating a conformational change in another protein significantly expands the functional repertoire of amyloid. Moreover, the mechanism of Het-S cell killing may be similar to the mechanism by which some pathological amyloid proteins lead to the demise of post-mitotic tissue.

## Abstract 2

Primers provide a concise introduction into an important aspect of biology highlighted by a current *PLOS Biology* research article.

## Prions and Amyloids in Sickness and in Health

**Figure 1:**
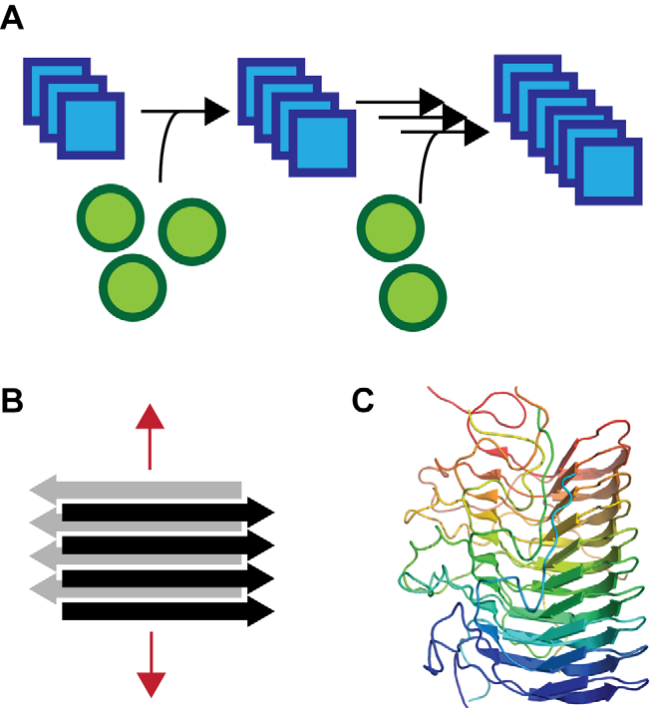
Prions are self-perpetuating protein structures that typically take the form of amyloid aggregates. A prion is a protein that can exist in two (or more) structures, one of which is self-replicating. (A) In the example shown, the prion or amyloid form of the protein (in blue) can convert the non-prion conformation (in green) to the prion form. Eventually, most of the non-prion protein is converted to the prion form. (B) Amyloid, which is a common prion structure, comprises a range of fibrous aggregates. Amyloid aggregates have related structures composed of stacked β-sheets (β-strands are represented by horizontal arrows), wherein the strands are oriented perpendicular to the fiber axis (indicated by the red arrow). Two antiparallel β-sheets are shown. (C) The Het-s prion is an amyloid aggregate that has a well-defined β-solenoid structure, as determined by solid-state NMR (PDB 2RNM) [25].

Prions are self-replicating protein structures that can be transferred between cells or animals by conversion of the native protein to the aggregated prion form (Figure 1A). Extracellular prions can be taken into prion-naïve cells, converting the soluble protein in the prion-naïve cell into an aggregated prion protein. Alternatively, cytoplasm between prion-harboring and prion-naïve cells can be exchanged, leading to additional prion formation.

Prions can be pathogenic or functional; for example, the human prion protein (PrP) is found as a membrane-tethered monomer in healthy individuals, but can spontaneously aggregate during vesicle trafficking and cause sporadic human prion diseases [1]. Human prion diseases can also be transmissible, e.g., when a healthy person comes into contact with surgical instruments contaminated by manipulating PrP-infected tissue. These introduced aggregates can induce aggregation of endogenous PrP and subsequent neurodegenerative disease in the human undergoing surgery [2].

Prions are fibrous aggregates with an amyloid quaternary structure consisting of β-strands arranged perpendicular to the long axis of the fiber [3]–[5] (Figure 1B). Amyloid aggregates are also a common feature of other human neurodegenerative diseases, such as Alzheimer's disease and Parkinson's disease. A large number of amyloid diseases, named after the amyloid aggregates common to these maladies, have been identified, and in each distinct amyloid disease, a different protein aggregates into an amyloid structure [6]. However, the amyloid quaternary structure can also play a physiological role in some organismal contexts; such functional amyloids have been identified in species ranging from bacteria to humans [7].

A number of functional amyloids exist in fungi, and remarkably, many of these are also prions. For example, in the yeast *Saccharomyces cerevisiae* the Sup35p protein can aggregate, resulting in the transmissible [*PSI^+^*] prion phenotype, thought to enhance phenotypic diversity by modulating the fidelity of translation termination [8]–[10]. Likewise, the Mod5 protein can aggregate, resulting in acquired drug resistance for cell survival under environmental stress [11]. A host of other *S. cerevisiae* prions have been discovered and are currently being characterized [12],[13].

## Prion-Mediated Fungal Strain Incompatibility

The coprophilic, filamentous fungus *P. anserina* harbors another example of a functional prion, [Het-s], one of the first examples of functional amyloid to be discovered [14],[15]. The *het-s* locus can encode one of two alleles: *Het-S* or *Het-s*. The *Het-*s-encoded protein, Het-s, is generally found in an aggregated or amyloid state in *P. anserina* strains in the wild (Figure 1C), and strains that have the prion phenotype are referred to as [Het-s] [16]. Because Het-s aggregates are passed to progeny, the [Het-s] phenotype is heritable. Strains that do not express Het-s instead express Het-S, which is generally found in a soluble state.

**Figure 2:**
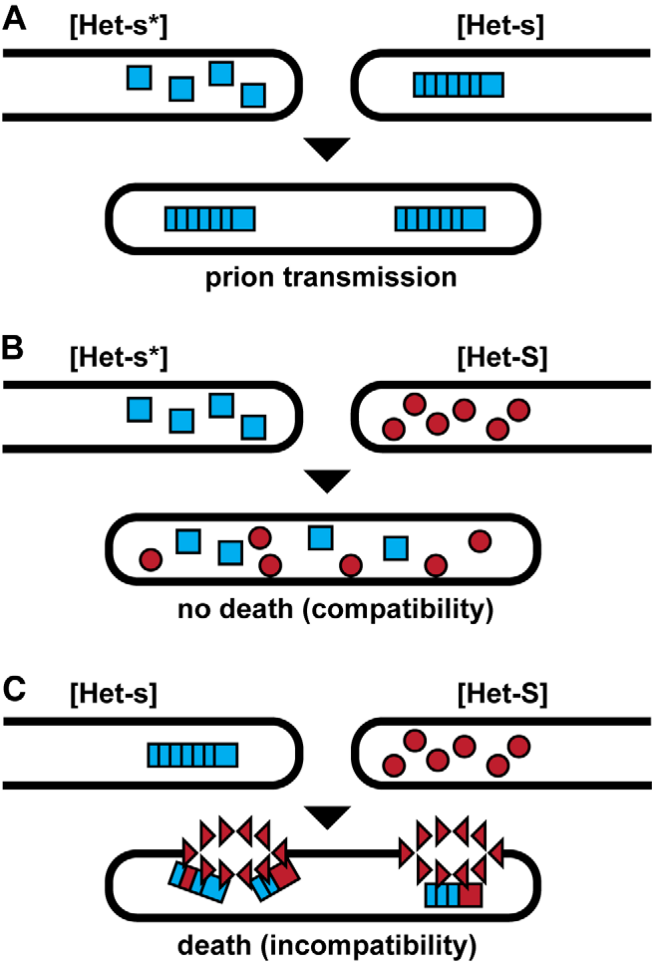
The *P. anserina* Het-s/Het-S locus mediates heterokaryon incompatibility. When two *P. anserina* mycelia fuse to form a heterokaryon, the Het-s/Het-S heterokaryon incompatibility system dictates whether the heterokaryon will be viable. The *P. anserina* Het-s locus has two alleles, *Het-s* and *Het-S*, meaning that a given mycelium expresses either the Het-s or Het-S protein. The Het-s protein generally adopts a prion structure and this form of Het-s is denoted [Het-s], whereas the soluble form is denoted [Het-s*]. Thus, there are three possible combinations of Het-s with Het-S. (A) A [Het-s*] strain can fuse with a [Het-s] strain to produce a viable heterokaryon in which the prion phenotype, [Het-s], is transmitted. (B) A [Het-s*] strain can fuse with a [Het-S] strain to produce a viable heterokaryon. (C) A [Het-s] strain can fuse with a [Het-S] strain to produce a nonviable heterokaryon. Het-s amyloid induced Het-S amyloid formation (indicated by red squares) leads to a conformational change in the Het-S HeLo domain that triggers intermolecular Het-S pore formation (indicated by red triangles).

When growing close together, two strains of *P. anserina* can fuse, mixing their cytoplasms to form a heterokaryon. Such fusion can confer a growth advantage and is a common feature among fungi [17]. However, heterokaryon formation can also facilitate the transfer of pathogens such as mycoviruses, senescence factors, or other parasites [14]. Thus, some fungi, including *P. anserina*, appear to have evolved so that heterokaryon formation generally leads to cell death. This phenomenon, called heterokaryon incompatibility, is mediated by the [Het-s] prion in *P. anserina* (Figure 2) [14]. During the incompatibility reaction, Het-S localizes to the plasma membrane, and it has been hypothesized that Het-s aggregates might mediate this change in localization by inducing a conformational change in the Het-S protein [18],[19]. However, the mechanism by which Het-s amyloid mediates heterokaryon incompatibility has been elusive.

## New Mechanistic Insights

In this issue of *PLOS Biology*, Seuring et al. present detailed insight into the mechanism by which aggregated Het-s converts the soluble Het-S protein into an integral membrane protein capable of disrupting the membrane, leading to a cell death program [20]. The authors primarily rely on elegant experiments in which they reconstitute the Het-s incompatibility reaction in liposomes *in vitro*. These assays show that upon exposure to aggregated Het-s, Het-S efficiently creates large defects in liposomal membranes. These defects are hypothesized to lead to the heterokaryon incompatibility response by controlled pore formation and cytotoxicity when they occur *in vivo*. Only small amounts of Het-s amyloid are required, suggesting that the aggregates act in a non-stoichiometric fashion to activate Het-S.

Because Het-S becomes membrane-associated in the presence of Het-s amyloid, the authors investigated how Het-S might change structure upon exposure to Het-s amyloid. Although Het-s and Het-S are highly homologous two-domain proteins, sharing 96% sequence identity, the small sequence differences produce large functional differences. Thus, despite each protein having a prion-forming C-terminal domain, only Het-s spontaneously exists as a prion. Each protein also has an N-terminal HeLo domain, which has previously been suggested to be important for heterokaryon incompatibility [14]. Seuring et al. now show that the Het-S HeLo domain can mediate membrane disruption in the presence of Het-s aggregates; moreover, merely heating Het-S can result in membrane disruption in the absence of aggregated Het-s. This is a crucial clue because it suggests that a conformational change in Het-S, induced either by heating or by binding to Het-s aggregates, renders Het-S capable of disrupting membrane integrity. In fact, such a conformational change had been proposed previously [18].

To probe the hypothesis that exposure of Het-S monomer to Het-s aggregates can cause a conformational change in the Het-S HeLo domain, the authors use a combination of solid-state nuclear magnetic resonance (NMR) and limited proteolysis. They find that, upon exposure to aggregated Het-s, the Het-S prion-forming domain adopts a mixed amyloid quaternary structure nearly identical to the one found in the Het-s aggregates. While not addressed in this paper, this also implies that Het-s amyloid fragmentation must be facile to enable co–Het-s/Het-S β-solenoid amyloid formation. The authors also outline very strong evidence that the Het-S HeLo domain becomes unstructured upon mixed Het-s/Het-S amyloid formation by their C-terminal domains.

The interpretation of the loss of HeLo domain structure in Het-S was unclear until the proteinase K data revealed membrane-mediated protection of the N-terminus upon Het-s amyloid-mediated conformational change. Bioinformatic analyses showed that the HeLo domain of Het-S, but not of Het-s, likely contains a hidden N-terminal transmembrane helix. The authors hypothesized that a conformational rearrangement of the Het-S HeLo domain induced by Het-s/Het-S C-terminal co-amyloidogenesis results in the exposure of this Het-S transmembrane helix which might then enable Het-S membrane association and subsequent membrane disruption. To confirm this hypothesis, the authors test several mutations in Het-s and Het-S [21]. They show that mutations that re-create the putative transmembrane helix in the HeLo domain of Het-s enable the mutant Het-s to disrupt liposomal membranes in a fashion analogous to wild type Het-S.

## A Pore-Forming Toxin?

Finally, the authors address the question of how a single transmembrane helix might induce membrane damage, leading to a cell death program. They hypothesized that Het-S transmembrane helices oligomerize to create a pore. Using multi-angle light scattering, the authors provide suggestive evidence that Het-S can oligomerize in the presence of detergents that simulate a membrane environment. Although pore formation seems likely at this juncture, further studies are required to fully elucidate the details of Het-S-mediated cytotoxicity. Moreover, while the authors invoke fiber fragmentation to allow Het-s/Het-S pore formation, we wonder whether a proteolysis step could liberate the pore-forming Het-S HeLo domain from the amyloid fiber.

Should the authors' hypothesis that Het-S forms pores in the fused cell membrane to mediate the heterokaryon incompatibility reaction prove correct, then the Het-S protein joins the family of pore-forming toxins. These are synthesized by a variety of organisms as defensive or offensive weapons to disrupt target membranes [22]. Pore-forming toxin proteins are synthesized and exported as soluble monomers, thereby minimizing damage to the cell generating the toxin. At the target membrane, the monomers undergo a conformational rearrangement that enables them to self-assemble into membrane-disrupting oligomers [22]. The data presented here suggest that Het-S meets the definition of a pore-forming toxin, regulated by Het-s/Het-S co-amyloidogenesis, though the structure of the Het-S oligomeric pore remains unknown.

## Broader Implications

The formation of pores that disrupt membrane structure is also thought to be important in the post-mitotic cell death observed in amyloid disease pathology [23],[24]: the hypothesis being that the process of amyloid fiber formation also leads to the formation of cytotoxic pores. Many pathological amyloid proteins generate intermediate oligomeric structures that are capable of acting as a pore [24]. Given that the Het-s/Het-S heterokaryon incompatibility reaction connects amyloid formation and cell death, many expected that the mechanism of Het-S cell killing might be amyloid-related pore formation. Surprisingly, Het-s/Het-S heterokaryon incompatibility is mediated by amyloid-induced conformational switching of the Het-S non-amyloid HeLo domain, exposing a transmembrane segment capable of subsequent pore formation, which probably involves helical assemblies. While the heterokaryon incompatibility mechanism does not appear to involve toxic oligomers thought to be important for pathological amyloids [23], the results in this paper suggest that amyloid fiber formation by a protein associated with pathology could exert cytotoxicity indirectly—by inducing a conformational change in another protein that could mediate aberrant pore formation in the membranes of post-mitotic tissues.

Characterization of functional amyloids in organisms ranging from bacteria to humans has revealed that the cross-β sheet amyloid structure can facilitate many different tasks, including templating small molecule chemical condensation reactions and providing structural support for biofilm formation [3],[7]. The data Seuring et al. present support the hypothesis that Het-s/Het-S amyloid co-aggregates function as a conformational switch, initiating a cascade of events leading to the *P. anserina* heterokaryon incompatibility reaction through a conformational change in the Het-S HeLo domain. This proposed mechanism constitutes the first structure-based hypothesis for any fungal incompatibility system. It will be very interesting to learn more about the mechanism of activated Het-S oligomerization/pore formation and what role Het-s/Het-S co-amyloidogenesis and/or proteolysis plays in the pore formation envisioned to explain heterokaryon-associated cytotoxicity.

## Abbreviations

NMR: nuclear magnetic resonance
PrP: human prion protein
